# Comparative Effects of Vitamin D Supplementation on Oxidative Stress in Relapsing–Remitting Multiple Sclerosis

**DOI:** 10.3390/cimb46120845

**Published:** 2024-12-14

**Authors:** Martyna Lis, Natalia Niedziela, Jowita Adamczyk-Zostawa, Jolanta Zalejska-Fiolka, Jarosław Szczygieł, Agata Sowa, Agata Świętek, Monika Adamczyk-Sowa

**Affiliations:** 1 Department of Neurology, Faculty of Medical Sciences in Zabrze, Medical University of Silesia, 40-055 Katowice, Poland; 2 Department of Ophthalmology, Faculty of Medical Sciences in Zabrze, Medical University of Silesia, 40-055 Katowice, Poland; 3 Department of Biochemistry, Faculty of Medical Sciences in Zabrze, Medical University of Silesia, 40-055 Katowice, Poland; 4 Silesia LabMed Research and Implementation Center, Medical University of Silesia in Katowice, 41-808 Zabrze, Poland

**Keywords:** multiple sclerosis, oxidative stress, 25(OH)D, vitamin D supplementation, antioxidants

## Abstract

Studies suggest that vitamin D (VitD) may reduce oxidative stress (OS) in multiple sclerosis (MS) patients. This study aimed to compare the effects of various VitD doses on OS in relapsing–remitting MS (RRMS). A 6-month supplementation was introduced using two doses of VitD: 2000 IU/day in the high-dose group (HD, *n* = 23) and 15,960 IU/month in the low-dose group (LD, *n* = 29). Significant differences in body weight, height, and age were found between groups. A significant increase in the level of VitD (25(OH)D) was noted in both groups (*p* < 0.01). A significant increase was observed in the levels of LF and MDA (*p* < 0.01) and a significant decrease in the concentrations of PSH (*p* < 0.01), CuZnSOD (*p* = 0.02), and TOS (*p* < 0.01). A significant positive correlation was observed between serum VitD and SOD (R = 0.38, *p* < 0.01) and MnSOD (R = 0.31, *p* < 0.05), as well as a significant negative correlation between serum VitD and MDA (R = −0.31, *p* = 0.05) at the beginning of the study. At the end of the study, a significant positive correlation was observed between serum VitD and SOD (R = 0.34, *p* < 0.05) and CuZnSOD (R = 0.51, *p* < 0.01). In RRMS patients, the VitD doses are probably insufficient to induce a beneficial effect on the pro- and antioxidant balance.

## 1. Introduction

Multiple sclerosis (MS) is a chronic disease associated with neurodegeneration and involving inflammation of the peripheral nervous system (PNS) and central nervous system (CNS), oxidative stress (OS), and the disruption of the blood–brain barrier (BBB), that leads to recurrent episodes of demyelination and axonal injury [1]. The disease affects individuals from early adulthood, and there are around 2.5 million people with MS worldwide. MS is one of the leading contributors to disability in young adults, and women are more often affected than men, which can be linked to the interplay of environmental, genetic, and epigenetic factors [2,3,4,5].

The disease can be categorized into several distinct phenotypes, primarily including relapsing–remitting MS (RRMS), characterized by episodes of relapses and followed by periods of partial or complete recovery; primary-progressive MS (PPMS), associated with progressive worsening of symptoms and disability; and secondary-progressive multiple sclerosis MS (SPMS), with deteriorating neurological function over time.

The pathogenesis of the disease is complex and associated with Th-cell-mediated neuroinflammation connected with hyperactivation of proinflammatory cells and macrophages, and the excessive generation of reactive nitrogen (RNS) and oxygen (ROS) species. OS is the result of a cellular imbalance between antioxidant defense and the production of free radicals that can modulate the permeability of the BBB, resulting in the exposure of the CNS to the pathological activity of immune cells [6,7,8]. The chronic production of cytokines and other immunological mediators, even during remission, contributes to the weakening of the adaptive antioxidant responses of the CNS [9]. In all stages of MS, OS contributes to changes in the white and grey matter, augmenting inflammation in the CNS and PNS [1]. In the early phase of MS, inflammation that increases the ROS level is the main pathological process that leads to demyelination, while the chronic stage of MS is mainly associated with structural and functional damage due to OS (neurodegeneration) [7,10,11]. Researchers indicate a significant increase in OS markers in MS individuals, with a decreased antioxidant defense compared to healthy controls [7,12,13,14,15,16].

Recent studies have shown significant changes in the pro- and antioxidant balance in patients with MS. Antioxidant defense can be assessed by determining the activity of various enzymes involved in neutralizing free radical activity, such as superoxide dismutase (SOD) and ceruloplasmin (CER). Additionally, non-enzymatic antioxidant status can be assessed by total antioxidant capacity (TAC), the level of protein sulfhydryl groups (SH), and lipofuscin (LF). In contrast, to assess pro-oxidant status, the level markers of lipid peroxidation like lipid hydroperoxides (LHP) and malondialdehyde (MDA) or total oxidative stress (TOS) can be determined [17].

It is presumed that MS occurs in individuals with genetic susceptibility under the influence of environmental factors that trigger an immune-mediated inflammatory response [18]. The incidence of MS increases with increasing latitude and lower exposure to sunlight, which prompted scientists to verify the role of vitamin D (VitD) in this group of patients [18,19]. Emerging research suggests that it may have a protective role against several autoimmune diseases, including MS. VitD deficiency is common worldwide, and recent studies stress that it may be associated not only with the development of MS but also with the severity of symptoms, a greater number of relapses, and a higher degree of disability [20]. In addition, VitD supplementation may reduce the disease’s activity [21] by reducing OS markers, not only in those with MS [22,23]. The influence of VitD on OS may be explained by its antioxidative properties and similarities in structure with natural antioxidants, such as cholesterol and ergosterol. In addition, by binding to its receptor (vitamin D receptor; VDR), VitD can upregulate the expression of selected antioxidant enzymes or may inhibit the secretion of ROS [22].

Many different meta-analyses have assessed the influence of VitD supplementation on OS markers. However, not much is known about this effect in MS patients. Furthermore, study findings are inconsistent in terms of the effect on pro- and antioxidant markers, as well as the dose of supplementation. Researchers underline that VitD supplementation may be used as an adjuvant therapy in diseases with high OS levels. However, more studies are needed, especially those associated with the duration of supplementation and an effective dose [24].

The study aimed to determine and compare the influence of supplementation with varying doses of VitD on OS parameters. We assessed the effect of a high dose (2000 IU/day) and a low dose (15,960 IU/month) VitD supplementation on the level of the selected antioxidant markers, such as SOD and its isoforms, i.e., manganese superoxide dismutase (MnSOD) and copper-zinc superoxide dismutase (CuZnSOD), CER, TAC, SH, and the concentration of selected markers of pro-oxidative status, including TOS, LHP, MDA, and LF in the serum of 52 patients with RRMS. The levels of VitD and OS markers were measured on the day of enrollment in the study and after 6 months of VitD supplementation.

## 2. Materials and Methods

### 2.1. Study Design and Patient Characteristics

This study was approved by the local Ethics Committee (approval number: PCN/0022/KB1/69/I/20/21) and followed the Helsinki Declaration. All the participants gave informed consent to participate in the study.

Patients with multiple sclerosis, diagnosed according to the McDonald criteria with revisions (2017) [25], who received treatment at the Multiple Sclerosis Center in Zabrze, were included in this study. The criteria for inclusion were age ≥ 18, disease-modifying therapy (DMT) for at least six months, and residency in the Silesia region (49°–50° N latitude). The exclusion criteria were the presence of neurological disorders other than MS, diseases that affect calcium–phosphate metabolism, MS phenotypes other than RRMS, any relapses occurring within the past six months, an Expanded Disability Status Scale (EDSS) score greater than 5, use of medications or dietary practices that affect calcium and phosphate homeostasis, VitD supplementation within the previous six months, travel to different climatic regions in the last six months, occupations involving underground work or exclusively staying at home, being in the menopausal stage, pregnancy or planned childbirth within six months, and breastfeeding. None of the patients discontinued or modified immunomodulatory therapy.

Of 309 initially selected MS patients from the Department of Neurology, 52 subjects who met the above criteria and reported for a follow-up were included. During the study, the subjects were supplemented with VitD for 6 months. The study participants were divided into two groups:

Low dose (LD)—comprised 29 patients who were administered calcifediol at a dosage of 15,960 IU/month (i.e., 530 IU/day) orally.

High dose (HD)—consisted of 23 MS patients who received cholecalciferol at a dosage of 2000 IU/day orally.

The study group included patients on DMT, such as dimethyl fumarate (*n* = 21), teriflunomide (*n* = 8), ocrelizumab (*n* = 6), interferon beta-1a (IFNβ-1a; *n* = 5), pegylated IFNβ-1a (*n* = 3), IFNβ-1b (*n* = 5), cladribine (*n* = 2), or fingolimod (*n* = 2).

### 2.2. Serum Analyses

Samples of blood were obtained on the day of enrollment in the study (October 2021–March 2022) and again after six months of VitD supplementation. The samples were centrifuged and frozen at −80 °C until further analysis. The laboratory assessments were performed at the beginning and after six months according to the producer’s protocol. The results were evaluated against the relevant standards for the specific testing methods. The concentrations were determined at the Silesia LabMed Research and Implementation Center, Medical University of Silesia, Poland.

To assess the level of 25(OH)D in the serum samples, an ELISA (sandwich enzyme-linked immunosorbent assay) kit (Immuniq, Żory, Poland) was used. According to the Endocrine Society Clinical Practice Guideline, the VitD status was determined depending on the level of 25(OH)D. VitD sufficiency was defined as 25(OH)D ≥ 30.0 ng/mL, insufficiency as 20–29.9 ng/mL, whereas deficiency was defined as 25(OH)D < 20.0 ng/mL, and severe deficiency as 25(OH)D < 10.0 ng/mL [26,27]. 25(OH)D < 30.0 ng/mL level was defined as hypovitaminosis D [28,29].

The concentration of LHP was evaluated based on the method of Södergren et al. [30] using xylenol orange. The serum concentration of MDA was assessed using the reaction with thiobarbituric acid according to Ohkawa et al. [31]. The method was modified by adding sodium sulfate and butylated hydroxytoluene, increasing the specificity of the method. The serum concentration of LF was determined according to the method of Tsuchida et al. [32,33]. The activity of SOD and its isoenzymes was determined according to Oyanagui [34]. The activity of CuZnSOD was determined as the difference between total SOD and MnSOD activity. Serum SH levels were determined in accordance with the method of Koster et al. [35] with the modification of the semi-automatic method. The concentration of CER in the blood serum was determined spectrophotometrically based on the Richterich method [36]. The TAC determination is based on the decolorization of oxidized ABTS under the influence of antioxidants occurring in the tested sera [37]. The absorbance readings were performed with different filters using the PerkinElmer VICTOR-X3 and the fluorescence intensity (Perkin Elmer LS45 spectrometer (PerkinElmer, Waltham, MA, USA)). The concentrations were calculated from the specific calibration curves.

### 2.3. Statistical Analysis

Data are expressed as median values along with interquartile range. To compare quantitative variables, the Wilcoxon test for dependent measurements was introduced. To estimate correlations between the parameters, Spearman’s Rho coefficient was used due to the non-normal distribution of the data, which was evaluated using a Q-Q plot. The analysis was conducted using the R language (version 4.1.2) in the RStudio (Posit, Boston, MA, USA) environment.

The nparLD test was used to calculate *p*-values for the effects of time, group, and the interaction between group and time, and to determine the relationship between VitD supplementation dosage and changes in the selected parameters over time. A significance level of *p* < 0.05 was established.

Additionally, a generalized estimating equation (GEE) model, adjusting for BMI and age, was performed to validate the obtained results (Supplementary Materials).

## 3. Results

The study group consisted of 52 individuals who met the inclusion criteria and were supplemented with vitamin D during the study period; 23 subjects (44.23%) were given a high dose of VitD, while 29 (55.77%) were on a low dose of VitD.

Females accounted for 63.46% of the participants. The median age [years] was 47 [40.0–55.0] in the LD group and 39.5 [34.5–49.8] in the HD group. A significant difference in age between LD and HD groups (*p* = 0.028) was noted. Significant differences between the groups were observed in body weight and height (*p* = 0.014 and *p* = 0.001, respectively). However, no statistical differences were found in the BMI (kg/m^2^) (*p* = 0.496). In the HD group, the median body weight [kg] was higher (74.0 [65.0–89.0]) compared to the LD group (65.0 [62.0–75.0]). BMI ≥ 25 kg/m^2^ (obesity) was observed in 31.03% of patients in the LD group and 34.78% of patients in the HD group. A total of 28.85% of all participants indicated that they smoke cigarettes, while 17.31% reported engaging in regular physical activity. The characteristics of the study group are given in Table 1.

A significant increase in the median 25(OH)D level [ng/mL] was observed in both groups (*p* < 0.01) and was significantly higher in the HD group (*p* = 0.01), in which the baseline concentration was lower. Due to VitD supplementation, the median concentration of VitD increased to a similar level in the HD and LD participants. Therefore, the rate of increase was higher in the HD group (*p* = 0.01). The median concentration of 25(OH)D during the study is presented in Table 2.

Most participants (71.2%) presented with hypovitaminosis D. After 6 months of supplementation, the serum level of VitD was still insufficient in 46.2% of participants. The status of VitD of the study participants based on the serum 25(OH)D concentration on the day of enrollment and after 6 months of VitD supplementation is given in Table 3.

None of the participants observed a relapse throughout the study. The median result in the EDSS (3.0 [2,3,4,5] in an HD and an LD group) did not alter significantly after completing VitD supplementation in both groups (*p* = 0.709).

**Figure 1:**
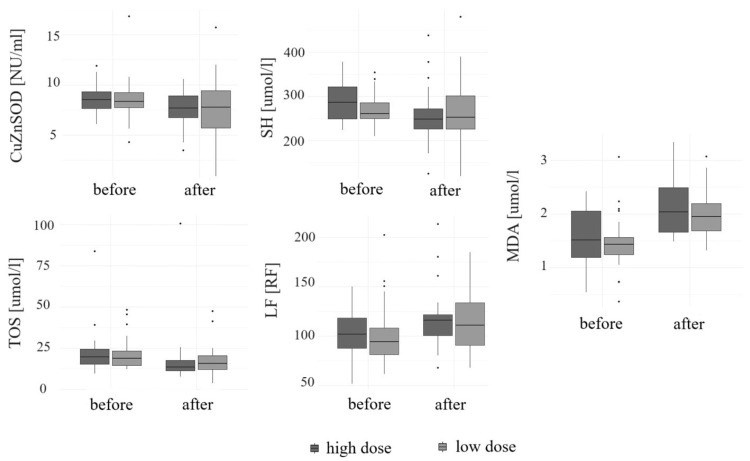
The concentrations of selected oxidative stress markers before and after six months of vitamin D supplementation depending on the supplemented dose.

A significant increase in the levels of LF [RF] and MDA [µmol/l] (both *p* < 0.01) was noted, regardless of the medication used (*p* = 0.78 and *p* = 0.55, respectively). An increase in the concentrations of CER [mg/dl] (*p* = 0.33), TAC [mmol/l] (*p* = 0.17), and MnSOD [NU/mL] (*p* = 0.08) was observed over the course of the study. However, it was not statistically significant. A significant decrease in the concentrations of SH [µmol/l] (*p* < 0.01), CuZnSOD [NU/mL] (*p* = 0.02), and TOS [µmol/l] (*p* < 0.01) was observed during supplementation, regardless of the preparation used (*p* = 0.18, *p* = 0.67, and *p* = 0.37, respectively). A decrease in the levels of LHP [µmol/l] (*p* = 0.08) and SOD [NU/mL] (*p* = 0.1) was found. However, it was not statistically significant. The concentrations of selected OS parameters at the beginning and the end of the study are given in Table 4. A change in the serum level of selected cytokines depending on the supplemented dose is given in Figure 1.

**Figure 2:**
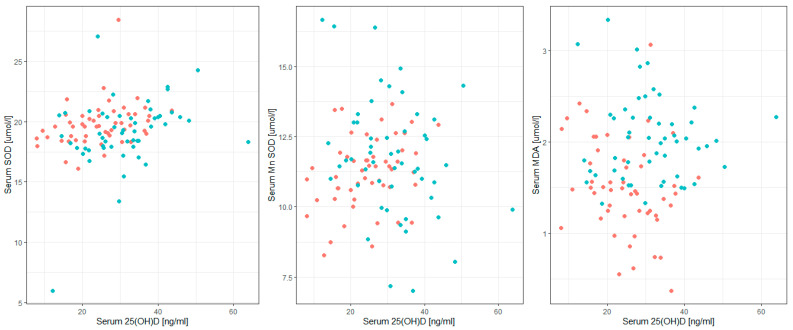
Correlations between serum levels of vitamin D and concentrations of selected oxidative stress markers before (red) and after (blue) vitamin D supplementation.

A significant positive correlation was observed between the serum concentrations of 25(OH)D and SOD (*p* < 0.01) and MnSOD (*p* < 0.05), while a significant negative correlation was found between serum 25(OH)D and MDA (*p* = 0.05) at the study onset. However, at the end of the study, a significant positive correlation was reported between the levels of 25(OH)D and SOD (*p* < 0.05) and CuZnSOD (*p* < 0.01) in serum. Correlations between the serum level of VitD and the concentration of the selected OS markers at two time points are given in Table 5. The correlations between serum levels of vitamin D and the concentrations of selected oxidative stress markers measured before and after vitamin D supplementation are presented in Figure 2.

The GEE model showed no significant interactions of age and BMI with the assessed parameters (Supplementary Materials).

## 4. Discussion

This paper is another part of our investigation in which we assessed the influence of the same low and high doses of VitD on the biomarkers of the disease activity, concluding that supplemented doses might be not sufficient to induce the desired anti-inflammatory results [38,39]. To the best of our knowledge, this is the first study evaluating the effect of VitD supplementation on the pro- and antioxidant balance in patients with RRMS.

As the study groups were heterogeneous in terms of age, height, and weight, a GEE model with adjustment for these factors was performed to confirm the obtained results and to exclude potential interactions with the analyzed parameters. It is suggested that age-related changes influencing VitD metabolism start at the age of 65 years [40] and the study group consisted of younger participants. It also seemed to be beneficial to introduce higher VitD doses in the HD group, due to the higher percentage of obese patients, who, according to recommendations, need higher VitD doses [41].

Most participants presented with VitD hypovitaminosis, which is in line with other reports [42] according to which patients with MS are at risk of reduced serum VitD levels, which we reported in a different study [43]. Interestingly, the effect of VitD on the disease course may be more apparent in patients with a lower baseline concentration of 25(OH)D [20]. After 6 months of supplementation, the serum levels of VitD were still insufficient and deficient in a high percentage of participants, which may indicate that the doses of supplementation were too low to achieve adequate concentration or the supplementation period was too short in those with a lower baseline level of 25(OH)D.

Markers of OS might be used as an essential tool in the assessment of the disease status in patients with MS. SOD is a major antioxidant enzyme that catalyzes the conversion of superoxide anions into oxygen and hydrogen peroxide, thereby controlling the levels of different free radicals and restricting their potential toxicity [44]. There are three different isoforms of SOD, i.e., cytoplasmic Cu/ZnSOD (SOD1), mitochondrial MnSOD (SOD2), and extracellular Cu/ZnSOD (SOD3) [45]. Adamczyk-Sowa et al. observed differences in the concentration of SOD isoforms in the group of RRMS patients who had higher levels of MnSOD in the serum and cerebrospinal fluid (CSF) and reduced levels of CuZnSOD in the CSF compared to healthy controls [46]. In fact, the level of SOD in patients with MS is lower than in healthy individuals [14,16]. However, Bizoń et al. reported that they found no significant differences in the SOD activity between RRMS individuals using different immunomodulatory drugs and healthy controls, which might have been due to effective DMT [47]. Additionally, some proinflammatory cytokines that are elevated not only in MS [8] but also in other conditions, such as COVID-19 infection [48], may potentially increase the activity of MnSOD [46]. Knowledge about the influence of VitD supplementation on SOD3 is limited, although studies have shown that VitD supplementation alone had little effect on the activity of SOD. A low synergistic effect was reported when VitD supplementation was combined with elastic resistance training in 40 healthy men [49]. In their 12-week randomized controlled clinical trial on individuals with type 2 diabetes mellitus (DM2), Shab-Bidar et al. also evaluated the influence of a low-dose supplementation of VitD with a yogurt drink containing calcium (fortified with 1000 IU VitD/day) compared to a placebo. They found an improvement in VitD status but not in the activity of SOD in the intervention group [50].

According to the researchers, the assessment of the SH level is also a good indicator of OS [51] being an important antioxidant system. The serum concentration of total SH groups is decreased in RRMS patients compared to healthy individuals, which may be explained by greater nitrosylation of SH groups and excessive redox-dependent changes. Additionally, it was noticed that the level of SH was lower during relapse but increased during corticosteroid therapy [9]. However, Stojanovic et al. found that the reduction in OS due to IFN therapy increased serum total SH groups in RRMS patients [52]. In our study, the decrease in the SH level might be attributed to the progression of the disease over time and the increase in OS. Other studies reported a negative correlation between the concentration of SH and the disease duration [53]. Furthermore, in their study on healthy children, Mertoglu et al. found that severe VitD deficiency was associated with impaired thiol/disulfide homeostasis and caused increased oxidation of proteins, which could not be reversed by external supplementation of VitD [54].

It was found that the concentration of pro-oxidative MDA, which can be used as an indicator of lipid peroxidation, was elevated in the serum of patients with RRMS [14,53,55]. Moreover, in the group of untreated patients, the level of MDA was significantly higher compared to patients on DMT, such as IFN [1]. However, some studies confirmed that supplementation with VitD might reduce the levels of MDA. In their randomized placebo-controlled study on 53 RRMS patients, Kouchaki et al. assessed the effects of ω-3 fatty acids and VitD co-supplementation (50,000 IU/biweekly) twice daily for 12 weeks. The results showed that in the supplementation group, the concentrations of serum high-sensitivity C-reactive protein (hs-CRP) and MDA decreased, and the levels of TAC and glutathione (GSH) significantly increased []. Other studies also confirmed a positive effect of supplementation on the selected OS parameters in different clinical conditions [19,56,57,58]. In their meta-analysis, Sepidarkish et al. found that the levels of TAC and GSH, which reflect an antioxidant defense, were increased, while the level of MDA decreased due to VitD supplementation compared to the placebo group [22]. The above results are not in line with our findings, which may result from lower doses of VitD. Sepidarkish et al. also noticed that the outcome depended on the dose of VitD, with the best results ranging from 100,000 to 200,000 IU per month [22].

Exhibiting pro-oxidant activity, LF can be induced by incomplete myelin digestion and disturbances in the lysosomal pathway, which involves phagocytosis of cellular material as a key mechanism leading to the accumulation of LF [59]. This may lead to a higher generation of ROS, sensitizing cells to oxidative injury through the destabilization of lysosomes [60]. Perivascular deposition of LF is an abnormality in white matter in MS individuals [61]. Accumulation of LF is a hallmark of aging and its increase in our study may be time-dependent. This finding is not in line with some reports stating antioxidants may reduce the accumulation of LF in the mitochondria of senescent cells [62]. Unfortunately, little is known about the accumulation of LF in MS.

In general, the evaluation of the overall antioxidant ability and OS can be performed by the assessment of the levels of individual antioxidants and pro-oxidants or by determining TAC and TOS, respectively. TOS reflects the general level of OS in the sample; hence, the decrease in TOS observed in our study may indicate effective DMT or suggest possible changes in the concentration of other OS parameters, which were not determined in our research. Our results clearly show that the evaluation of particular mediators more accurately reflects changes in the pro- and antioxidant homeostasis in the serum of RRMS patients. TOS and TAC may be useful for general assessment, whereas specific changes of particular parameters and their interactions may not be seen, which was reported by other researchers [47,50,58].

Our study did not show a clear positive influence of VitD supplementation on pro- and antioxidative stress balance, which may indicate that such low doses of VitD do not contribute to the reduction in OS, as was found in other studies. This may raise a suspicion that the changes in these parameters are associated with different factors. Our results may indicate that during RRMS, oxidative processes may be more pronounced over time, while antioxidant defense in patients chronically exposed to OS is not entirely adequate. Ljubisavljevic et al. found that as the disease progresses, exposure to oxidative damage is prolonged, which may change the response of different mechanisms preventing OS and even cause irreversible inactivation [9]. As already mentioned, the level of OS is higher during the active progression of MS compared to remission periods, or healthy controls [14]. In addition, during oxidative injury and neurodegeneration, the expression of antioxidant enzymes and their activity in MS is diversified, which was found in our study [47]. Another contributing factor might be a time-related progression and disease activity (undiagnosed relapses, changes in MRI activity) that may be responsible for the induction of OS.

Furthermore, serum samples were collected mainly in the autumn–winter period commonly associated with frequent infections, including COVID-19, which influence the level of different cytokines contributing to inflammatory changes, thus increasing the level of OS in cells [63]. Furthermore, many factors contribute to OS, such as age, gender, lifestyle, and exposure to toxic substances, such as those in polluted air [64]. In addition, the assessed markers are involved in various processes and could be associated with other clinical conditions in our study participants.

Importantly, the observed correlations between Vit D and selected OS markers may indicate a possible beneficial effect of VitD, which could be more evident during the deficiency of VitD, due to the fact that these observations were time-related, and may indicate that the antioxidative actions of VitD could depend on the serum 25(OH)D status. When the VitD status is physiological, many OS-related actions are downregulated, while suboptimal levels upregulate OS-related damage and apoptosis of neurons, which is in line with the finding that hypovitaminosis D may predispose individuals to MS and could have a role in its pathogenesis [5,18,19,65,66]. Dhas et al. also observed that significant differences in the MDA level between healthy controls and patients with DM2 were observed depending on the VitD status (MDA was significantly different between both groups only in insufficiency and moderate deficiency categories, but not in severe deficiency and sufficiency categories) [67]. Their findings are not in line with other studies in which MDA levels in different health conditions could be inversely correlated with the concentration of VitD [68] and the findings that the supplementation could contribute to the reduction in OS expressed by a decrease in the concentration of MDA [19,22,56].

## 5. Conclusions

Vitamin D plays an essential role in combating OS because of its antioxidant abilities. To date, very few studies have investigated the link between VitD supplementation and OS in RRMS patients.

Our study shows that, among RRMS patients, the doses of introduced VitD are probably too low to induce noticeable beneficial effects on the pro- and antioxidant homeostasis. A significant increase in the concentration of pro-oxidative LF and MDA was observed. When considering antioxidant defense, a decrease was noted in the SH and CuZnSOD levels, which may be associated with the influence of confounding factors other than VitD.

Therefore, the overall impact of VitD supplementation on oxidative stress in this population remains uncertain, warranting further investigation. Future studies should aim to identify effective supplementation strategies that could potentially reduce oxidative stress and align with individual VitD statuses, particularly given the prevalence of hypovitaminosis D among MS patients. Regular assessment of VitD level could aid in optimizing treatment strategies and improving health outcomes.

## 6. Limitations

One of the limitations of the study was the small size of the study group. Additionally, there was no control group and we did not introduce a placebo. This is why our results should be treated as preliminary. What’s more, the participants were taking various DMTs, which could contribute to different immune responses to VitD supplementation. However, there are no studies confirming that DMT could impact the VDR receptor through which VitD exerts its action. Following the assessment of OS parameters, it would be useful to measure basic serum markers of inflammation to exclude the influence of other confounding factors. In addition, dietary habits in the study group should be further analyzed.

## Figures and Tables

**Table 1:** The characteristics of the study group depending on the dose of vitamin D supplementation.

	HD ^1^ Group			LD ^2^ Group			*p* Value
Variable	Median	q1	q3	Median	q1	q3	
Age [years]	39.5	34.5	49.8	47.0	40.0	55.0	0.028
Weight [kg]	74.0	65.0	89.0	65.0	62.0	75.0	0.014
Height [m]	1.8	1.7	1.8	1.7	1.6	1.8	0.001
BMI ^3^ [kg/m^2^]	23.6	22.8	27.1	23.4	22.1	26.8	0.496
Age at diagnosis [years]	34.0	26.0	47.0	33.5	28.5	43.8	0.626
Disease duration [years]	6.0	4.0	12.0	8.0	3.0	15.8	0.215
Age at first symptoms [years]	31.0	24.5	43.0	32.0	27.5	39.3	0.601
Duration of immunomodulatory treatment [months]	53.0	42.0	104.0	80.5	41.3	132.8	0.866
The number of MS ^4^ relapses	2.0	1.0	4.0	3.0	1.0	5.3	0.240
Time from the last relapse [months]	56.0	31.5	70.0	55.0	33.5	82.0	0.724
The number of MS relapses treated with GCs ^5^	2.0	1.0	3.5	2.0	1.0	5.5	0.125
Time from the last administration of GCs [months]	58.0	37.0	77.0	55.0	31.5	74.5	0.652
EDSS ^6^ [score]	3.0	2.5	3.5	3.0	2.5	3.5	0.746
Time from smoking initiation [years]	20.0	10.0	27.5	20.0	18.8	27.5	0.396
The number of cigarettes/day	15.0	11.3	18.8	12.5	10.0	18.8	0.622
Amount of physical activity [min/week]	165.0	67.5	292.5	120.0	60.0	210.0	0.368

^1^ HD—high dose of vitamin D supplementation, ^2^ LD—low dose of vitamin D supplementation, ^3^ BMI—body mass index, ^4^ MS—multiple sclerosis, ^5^ GCs—glucocorticosteroids, ^6^ EDSS—expanded disability status scale.

**Table 2:** Serum concentrations of 25(OH)D before and after vitamin D supplementation.

		Before			After			GE ^4^ (p)	TE ^5^ (p)	GETE ^6^ (p)
Variable	Group	Median	q1	q3	Median	q1	q3			
25(OH)D ^1^ [ng/mL]	HD ^2^	23.023	15.578	25.760	29.819	24.937	38.064	0.19	0.00	0.01
	LD ^3^	28.318	20.644	32.232	30.837	25.382	36.789			

^1^ 25(OH)D—25-Hydroxyvitamin D, ^2^ HD—high dose of vitamin D supplementation, ^3^ LD—low dose of vitamin D supplementation, ^4^ GE—group effect, ^5^ TE—time effect, ^6^ GETE—group effect×time effect.

**Table 3:** The status of vitamin D in the study participants depending on the serum concentrations of 25(OH)D before and after supplementation.

Vitamin D Status	Before		After	
	n	[%]	n	[%]
Severe deficiency [0–9.9 ng/mL]	3.0	5.8	0.0	0.0
Deficiency [10–19.9 ng/mL]	12.0	23.1	6.0	11.5
Insufficiency [20–29.9 ng/mL]	22.0	42.3	18.0	34.6
Sufficiency [30–49.9 ng/mL]	15.0	28.8	26.0	50.0
High concentration [≥50 ng/mL]	0.0	0.0	2.0	3.9
Hypovitaminosis D [<30 ng/mL]	37.0	71.2	24.0	46.2

**Table 4:** Serum concentrations of 25(OH)D and the selected oxidative stress markers before and after vitamin D supplementation.

		Before			After			GE ^13^ (p)	TE ^14^ (p)	GETE ^15^ (p)
Variable	Group	Median	q1	q3	Median	q1	q3			
SOD ^3^ [NU/mL]	HD ^1^	19.599	18.538	20.348	19.188	18.288	20.490	0.95	0.10	0.24
	LD ^2^	19.590	18.820	20.871	19.070	17.811	20.387			
CuZnSOD ^4^ [NU/mL]	HD	8.544	7.657	9.323	7.702	6.747	8.914	0.84	0.02	0.67
	LD	8.365	7.750	9.241	7.793	5.708	9.424			
MnSOD ^5^ [NU/mL]	HD	11.026	10.446	11.633	11.699	11.191	13.069	0.88	0.08	0.14
	LD	11.441	10.705	12.019	11.484	10.331	12.463			
CER ^6^ [mg/dL]	HD	40.684	34.482	44.910	41.231	35.548	45.070	0.74	0.33	0.45
	LD	38.155	34.805	43.868	40.129	35.020	46.830			
SH ^7^ [µmol/L]	HD	286.720	249.121	321.574	248.770	226.282	271.955	0.78	0.00	0.18
	LD	261.200	250,000	285.600	253,000	226.144	301.200			
TAC ^8^ [mmol/L]	HD	1.001	0.925	1.080	1.070	0.976	1.145	0.99	0.17	0.25
	LD	1.029	0.953	1.122	1.054	0.935	1.121			
TOS ^9^ [µmol/L]	HD	19.758	15.268	24.422	13.584	11.282	17.581	0.49	0.00	0.37
	LD	18.905	14.469	23.263	15.783	12.088	20.426			
LHP ^10^ [µmol/L]	HD	5.989	4.281	8.918	4.347	3.610	5.721	0.55	0.08	0.45
	LD	5.779	4.251	8.704	5.360	3.957	7.092			
LF ^11^ [RF]	HD	102.070	87.748	118.328	116.219	100.659	121.676	0.48	0.00	0.78
	LD	94.479	81.370	108.213	111.197	90.793	133.737			
MDA ^12^ [µmol/L]	HD	1.518	1.190	2.054	2.041	1.661	2.491	0.17	0.00	0.55
	LD	1.437	1.244	1.564	1.954	1.687	2.194			

^1^ HD—high dose of vitamin D supplementation, ^2^ LD—low dose of vitamin D supplementation, ^3^ SOD—superoxide dismutase, ^4^ CuZnSOD—copper-zinc superoxide dismutase, ^5^ MnSOD—manganese superoxide dismutase, ^6^ CER—ceruloplasmin, ^7^ SH—protein sulfhydryl groups, ^8^ TAC—total antioxidant capacity, ^9^ TOS—total oxidative stress, ^10^ LHP—lipid hydroperoxides, ^11^ LF—lipofuscin, ^12^ MDA—malondialdehyde, ^13^ GE—group effect, ^14^ TE—time effect, ^15^ GETE—group effect×time effect.

**Table 5:** Correlations between serum levels of vitamin D and the concentrations of selected oxidative stress markers before and after supplementation of vitamin D.

Time	Variable 1	Variable 2	R	*p* Value
Before	25(OH)D ^1^	SOD ^2^	0.38	0.00550
		MnSOD ^3^	0.31	0.02670
		MDA ^4^	−0.31	0.02450
After	25(OH)D	SOD	0.34	0.01500
		CuZnSOD ^5^	0.51	0.00013

^1^ 25(OH)D—25-Hydroxyvitamin D, ^2^ SOD—superoxide dismutase, ^3^ MnSOD—manganese superoxide dismutase, ^4^ MDA—malondialdehyde, ^5^ CuZnSOD—copper-zinc superoxide dismutase.

## Data Availability

The data presented in this study are available on request from the corresponding author (data are not publicly available due to privacy and ethical restrictions).

## Supplementary Materials

The following supporting information can be downloaded at: https://www.mdpi.com/article/10.3390/cimb46120845/s1, File S1: GEE model.
