# Supplementary material for: Comparative Effects of Vitamin D Supplementation on Oxidative Stress in Relapsing–Remitting Multiple Sclerosis
Source: Curr Issues Mol Biol. 2024 Dec 14;46(12):14119–31. doi: 10.3390/cimb46120845 (PMC11674365; doi:10.3390/cimb46120845)
Supplement: Supplementary file 1 [file cimb-46-00845-s001.zip › cimb-3320789-supplementary.pdf]

File S1. GEE model.

```
[1]]

Call:
geeglm(formula = `25OHD` ~ Time + Supplemented_dose +
    BMI, data = ., id = Lp)

 Coefficients:
                  Estimate Std.err  Wald Pr(>|W|)
(Intercept)         15.414   3.541 18.95  1.3e-05 ***
Time                 6.635   1.788 13.78  0.00021 ***
Supplemented_dose    3.030   1.783  2.89  0.08915 .
BMI                  0.031   0.103  0.09  0.76279
---
Signif. codes:  0 '***' 0.001 '**' 0.01 '*' 0.05 '.' 0.1 ' ' 1

Correlation structure = independence
Estimated Scale Parameters:

            Estimate Std.err
(Intercept)     83.1    12.6
Number of clusters:   104  Maximum cluster size: 1

[[2]]

Call:
geeglm(formula = ALCAM ~ Time + Supplemented_dose + BMI, data = .,
    id = Lp)
```

Coefficients:

```
                 Estimate  Std.err  Wald Pr(>|W|)
(Intercept)        2.77463  0.74744 13.78  0.00021 ***
Time              -0.22662  0.30850  0.54  0.46260
Supplemented_dose   -0.08786  0.30970  0.08  0.77666
BMI               0.00388  0.01825  0.05  0.83168
---
Signif. codes:  0 ‘***’ 0.001 ‘**’ 0.01 ‘*’ 0.05 ‘.’ 0.1 ‘ ’ 1

Correlation structure = independence
Estimated Scale Parameters:

           Estimate Std.err
(Intercept)     2.47   0.546
Number of clusters:   104  Maximum cluster size: 1

[[3]]

Call:
geeglm(formula = CXCL16 ~ Time + Supplemented_dose + BMI, data = .,
    id = Lp)

 Coefficients:
                 Estimate Std.err Wald Pr(>|W|)
(Intercept)        0.00340 0.21229 0.00    0.987
Time               0.11171 0.08458 1.74    0.187
Supplemented_dose    0.01637 0.08505 0.04    0.847
BMI               0.00837 0.00472 3.14    0.076 .
---
Signif. codes:  0 ‘***’ 0.001 ‘**’ 0.01 ‘*’ 0.05 ‘.’ 0.1 ‘ ’ 1
```

Correlation structure = independence

Estimated Scale Parameters:

```
            Estimate Std.err
(Intercept)    0.186   0.049
```

Number of clusters: 104 Maximum cluster size: 1

[[4]]

Call:

geeglm(formula = PTX_3 ~ Time + Supplemented_dose + BMI, data = .,

  id = Lp)

Coefficients:

```
                  Estimate Std.err  Wald Pr(>|W|)
(Intercept)         2448.5   385.3 40.39  2.1e-10 ***
Time                 271.9   139.0  3.83    0.050 .
Supplemented_dose     14.9   141.0  0.01    0.916
BMI                  -19.0    10.9  3.02    0.082 .
---
Signif. codes:  0 ‘***’ 0.001 ‘**’ 0.01 ‘*’ 0.05 ‘.’ 0.1 ‘ ’ 1
```

Correlation structure = independence

Estimated Scale Parameters:

```
            Estimate Std.err
(Intercept)   502143   60314
```

Number of clusters: 104 Maximum cluster size: 1

[[5]]

Call:

geeglm(formula = IL_1RA ~ Time + Supplemented_dose + BMI, data = .,

id = Lp)

Coefficients:

	Estimate	Std.err	Wald	Pr(>|W|)	
(Intercept)	1145.3	556.1	4.24	0.039	*
Time	-45.6	184.9	0.06	0.805	
Supplemented_dose	42.1	181.7	0.05	0.817	
BMI	12.4	17.5	0.50	0.479	

Signif. codes: 0 ‘***’ 0.001 ‘**’ 0.01 ‘*’ 0.05 ‘.’ 0.1 ‘ ’ 1

Correlation structure = independence

Estimated Scale Parameters:

	Estimate	Std.err
(Intercept)	888867	198728

Number of clusters: 104 Maximum cluster size: 1

[[6]]

Call:

geeglm(formula = OPG ~ Time + Supplemented_dose + BMI, data = .,

id = Lp)

Coefficients:

	Estimate	Std.err	Wald	Pr(>|W|)	
(Intercept)	3.4967	0.7406	22.29	2.3e-06	***
Time	1.2036	0.2796	18.53	1.7e-05	***
Supplemented_dose	0.4184	0.2815	2.21	0.14	

```
BMI                        0.0266  0.0205  1.69     0.19
---
Signif. codes:  0 ‘***’ 0.001 ‘**’ 0.01 ‘*’ 0.05 ‘.’ 0.1 ‘ ’ 1

Correlation structure = independence
Estimated Scale Parameters:

            Estimate Std.err
(Intercept)     2.03   0.379
Number of clusters:   104  Maximum cluster size: 1

[[7]]

Call:
geeglm(formula = `25OHD` ~ Time + Supplemented_dose +
    Age_years, data = ., id = Lp)

 Coefficients:
                  Estimate Std.err  Wald Pr(>|W|)
(Intercept)        11.7035  4.6411  6.36  0.01168 *
Time                6.6119  1.7872 13.69  0.00022 ***
Supplemented_dose   3.0862  1.7644  3.06  0.08026 .
Age_years           0.0950  0.0851  1.25  0.26383
---
Signif. codes:  0 ‘***’ 0.001 ‘**’ 0.01 ‘*’ 0.05 ‘.’ 0.1 ‘ ’ 1

Correlation structure = independence
Estimated Scale Parameters:

            Estimate Std.err
(Intercept)     81.4    12.8
```

Number of clusters: 102 Maximum cluster size: 1

[[8]]

Call:

geeglm(formula = ALCAM ~ Time + Supplemented_dose + Age_years,

data = ., id = Lp)

Coefficients:

	Estimate	Std.err	Wald	Pr(>|W|)	
(Intercept)	1.6844	0.6758	6.21	0.013	*
Time	-0.2757	0.3056	0.81	0.367	
Supplemented_dose	-0.1818	0.3256	0.31	0.577	
Age_years	0.0287	0.0167	2.96	0.085	.

Signif. codes: 0 ‘***’ 0.001 ‘**’ 0.01 ‘*’ 0.05 ‘.’ 0.1 ‘ ’ 1

Correlation structure = independence

Estimated Scale Parameters:

	Estimate	Std.err
(Intercept)	2.38	0.476

Number of clusters: 102 Maximum cluster size: 1

[[9]]

Call:

geeglm(formula = CXCL16 ~ Time + Supplemented_dose + Age_years,

data = ., id = Lp)

Coefficients:

```
                 Estimate  Std.err Wald Pr(>|W|)
(Intercept)                  0.000109 0.247074 0.00     1.00
Time                         0.123824 0.084037 2.17     0.14
Supplemented_dose            0.035615 0.080134 0.20     0.66
Age_years                    0.003884 0.003294 1.39     0.24

Correlation structure = independence
Estimated Scale Parameters:

            Estimate Std.err
(Intercept)     0.18  0.0492
Number of clusters:   102  Maximum cluster size: 1

[[10]]

Call:
geeglm(formula = PTX_3 ~ Time + Supplemented_dose + Age_years,
    data = ., id = Lp)

 Coefficients:
                 Estimate Std.err  Wald Pr(>|W|)
(Intercept)                  2262.54  357.71 40.01  2.5e-10 ***
Time                         291.86  140.45  4.32    0.038 *
Supplemented_dose             72.32  157.09  0.21    0.645
Age_years                    -8.38    6.52  1.65    0.199
---
Signif. codes:  0 ‘***’ 0.001 ‘**’ 0.01 ‘*’ 0.05 ‘.’ 0.1 ‘ ’ 1

Correlation structure = independence
Estimated Scale Parameters:
```

```
            Estimate Std.err
(Intercept)   502992   68416
Number of clusters:   102  Maximum cluster size: 1

[[11]]

Call:
geeglm(formula = IL_1RA ~ Time + Supplemented_dose + Age_years,
    data = ., id = Lp)

 Coefficients:
                   Estimate Std.err  Wald Pr(>|W|)
(Intercept)                2467.03  426.30 33.49  7.2e-09 ***
Time                       -44.04  176.23  0.06  0.80266
Supplemented_dose     238.82  183.31  1.70  0.19264
Age_years                  -25.34    6.88 13.57  0.00023 ***
---
Signif. codes:  0 '***' 0.001 '**' 0.01 '*' 0.05 '.' 0.1 ' ' 1

Correlation structure = independence
Estimated Scale Parameters:

            Estimate Std.err
(Intercept)   791979  186132
Number of clusters:   102  Maximum cluster size: 1

[[12]]

Call:
geeglm(formula = OPG ~ Time + Supplemented_dose + Age_years,
    data = ., id = Lp)
```

Coefficients:

	Estimate	Std.err	Wald	Pr(>|W|)	
(Intercept)	2.3709	0.5827	16.56	4.7e-05	***
Time	1.2150	0.2545	22.80	1.8e-06	***
Supplemented_dose	0.4340	0.2508	2.99	0.08358	.
Age_years	0.0377	0.0098	14.83	0.00012	***

Signif. codes: 0 ‘***’ 0.001 ‘**’ 0.01 ‘*’ 0.05 ‘.’ 0.1 ‘ ’ 1

Correlation structure = independence

Estimated Scale Parameters:

	Estimate	Std.err
(Intercept)	1.65	0.33

Number of clusters: 102 Maximum cluster size: 1
